# Postpartum Depression in the United States: A Systematic Review of Epidemiology, Risk Factors, and Contemporary Management With Emphasis on Emerging Therapeutic and Digital Care Models

**DOI:** 10.7759/cureus.109962

**Published:** 2026-05-31

**Authors:** Motunrayo T Ojo-Rowland, Okelue E Okobi, Ikenna C Madubuike, Rita Kimble, Akintunde C Akinboboye, Roseline Igbadumhe, Anulika Anyata, Keturah Yusuf, Wuraola R Awosan

**Affiliations:** 1 Department of Obstetrics and Gynaecology, Zaporozhye State Medical University, Zaporozhye, UKR; 2 Department of Family Medicine, Larkin Community Hospital Palm Springs Campus, Miami, USA; 3 Department of Medicine, Avalon University School of Medicine, Willemstad, CUW; 4 Carey Business School, Johns Hopkins University, Baltimore, USA; 5 Department of Research and Development, University of Maryland, Laurel, USA; 6 Department of Emergency Medicine, University of Medical Sciences Teaching Hospital, Ondo, NGA; 7 Department of Psychology, Alaska Native Medical Center, Anchorage, USA; 8 Department of Community Medicine, Alex Ekwueme Federal University Ndufu-Alike, Abakaliki, NGA; 9 Department of Family Medicine, Baptist Memorial Hospital, Southaven, USA; 10 Department of Medicine, Ternopil National Medical University, Ternopil, UKR

**Keywords:** digital interventions, epidemiology, health disparities, maternal mental health, postpartum depression, telemedicine

## Abstract

Postpartum depression (PPD) is a significant public health concern in the United States, affecting many women after childbirth and contributing to adverse maternal and infant outcomes. This study aimed to synthesize current evidence on the epidemiology, risk factors, and management of PPD, with particular attention to emerging therapeutic and digital care approaches. A systematic search of major databases identified 25 eligible studies published between 2010 and 2026. The findings indicate that PPD remains prevalent, with disparities strongly associated with race, socioeconomic status, and access to care. Risk factors are multifactorial and include prior mental illness, low social support, unintended pregnancy, and psychosocial stressors, with growing evidence also pointing to genetic contributions. Symptoms may emerge at different stages of the postpartum period, underscoring the importance of ongoing screening rather than a single assessment. While psychotherapy and antidepressant therapy remain the foundation of treatment, newer strategies such as telehealth, mobile health applications, and wearable technologies show promise in improving early detection and access to care. Overall, addressing PPD requires integrated, equitable, and patient-centered approaches to improve maternal and child health outcomes.

## Introduction and background

Postpartum depression (PPD) is a prevalent yet serious mental health condition experienced by women following childbirth, with significant consequences for maternal well-being, infant development, and family functioning [1,2]. In the United States, PPD is recognized as an important public health issue, affecting approximately 10%-20% of new mothers, depending on population characteristics, screening methods, and timing of assessment [3]. Despite increased awareness and the availability of screening tools, PPD remains underdiagnosed and undertreated, particularly among socioeconomically disadvantaged and ethnic minority populations [4]. This underdiagnosis can result in delayed treatment, worsening symptom severity, impaired mother-infant bonding, and long-term adverse developmental outcomes in children. Barriers contributing to these disparities include limited access to healthcare services, stigma surrounding mental health, cultural differences in symptom recognition, and structural inequities within healthcare systems.

Postpartum depression arises due to multiple interacting risk factors, including significant recent findings indicating previous mental illness, low level of social support, social stresses, and the need for improved physical health [5]. Recent clinical reviews confirm that psychological stressors, such as previous episodes of depression or anxiety, financial difficulties, relationship problems, unplanned pregnancies, and lack of social support, remain the primary causes of illness during the postpartum phase [6,7]. The association of stress and depressive symptoms has been mediated by social support, which acts as a buffer between high exposure to stress and the risk of developing postpartum depression [8].

Postpartum depression may present with a range of symptoms, including prolonged sadness, anxiety, fatigue, and reduced ability to carry out daily activities, persisting beyond the normal “baby blues” experienced after childbirth [9]. If left untreated, PPD can negatively affect maternal well-being, impair mother-infant bonding, and contribute to delays in the cognitive and emotional development of children [10]. These impacts highlight the importance of distinguishing PPD from transient postpartum mood changes and underscore the need for timely identification and appropriate intervention. Early recognition and treatment are essential to mitigate long-term adverse outcomes for both the mother and the child. Research has identified that genetic variation associated with the brain-derived neurotrophic factor (BDNF) valine with methionine at codon 66 (Val66Met) polymorphism is highly associated with postpartum depressive symptomatology, and environmental context (season of delivery) interacts with this genetic variation to influence the vulnerability to postpartum depression [11]. Additionally, lack of social support, intimate partner violence, financial instability, and previous mental health history can be psychosocial stressors that increase a woman’s risk of developing PPD [12].

Traditional approaches to treatment, such as psychotherapy and conventional antidepressant medications, continue to play an important role, while brexanolone is available for the treatment of severe postpartum depression but has limitations due to its high cost and cumbersome delivery method [13]. Emerging US research provides early indications that data collected from wearable devices may help clinicians identify women with postpartum depression sooner [14]. Therefore, while digital health interventions are beneficial, they should complement direct clinical follow-up, especially for women who are at increased risk or have severe symptoms [15].

Additionally, the development of technology to aid in the delivery of mental healthcare has caused a shift in the way care is being delivered [16]. Telehealth/telemedicine, mobile health applications, and inference-based cognitive behavioral therapy (iCBT) are all examples of ways to offer mental health treatment to individuals [17]. They have been integrated to fill the gaps in the care system created by barriers to using traditional means of obtaining care, such as a lack of childcare or being physically unable to make it to appointments [18]. Additionally, universal screening programs are widely used in improving the identification, treatment uptake, and clinical outcomes for perinatal depression [19].

The objective of this study is to systematically synthesize recent US-based evidence on the epidemiology and risk factors of postpartum depression and evaluate contemporary management approaches, including novel pharmacological therapies and digital care models. The focus of the review will be to inform and improve future clinical practice and provide a structure for future research on PPD.

## Review

Methods

Eligibility Criteria and Search Strategies

This systematic review was conducted as per Preferred Reporting Items for Systematic Reviews and Meta-Analyses (PRISMA) 2020 guidelines with an aim of ensuring transparency, reproducibility, and methodological rigor [20]. This review was not prospectively registered, and no formal protocol was published prior to study selection. The study carefully selected articles that are peer-reviewed, published between 2010 and 2026, with the final search conducted in March 2026. Search updates were performed to ensure inclusion of the most recent studies. A comprehensive database-specific search strategy was developed using Boolean operators and controlled vocabulary where applicable. For example, the PubMed search strategy included the following: (“postpartum depression” OR “postnatal depression” OR “maternal mental health”) AND (“risk factors” OR “social support” OR “mental health history” OR “digital health” OR “telehealth” OR “pharmacological treatment”) AND (“United States” OR “U.S. population” OR “national datasets”) AND (“screening” OR “treatment outcomes” OR “depression symptoms”). Similar strategies were adapted for Embase, Scopus, Web of Science, and Cochrane Library, as shown in Table 1.

**Table 1:** Overview of search strategy PRAMS: Pregnancy Risk Assessment Monitoring System, NHANES: National Health and Nutrition Examination Survey

Category	Details
Databases searched	PubMed, Scopus, Web of Science, Embase, Cochrane Library
Time frame	Studies published between 2010 and 2026 were included
Language	English only
Search strategy structure	The search strategy was structured using a combination of four key components: #1, #2, #3, and #4
#1 (Population)	Postpartum depression OR postnatal depression OR maternal mental health
#2 (Exposure/risk factors/intervention)	Risk factors OR social support OR mental health history OR digital health OR telehealth OR pharmacological treatment
#3 (Study context)	United States OR US population OR national datasets (e.g., PRAMS, NHANES)
#4 (Outcome)	Depression symptoms OR screening outcomes OR treatment outcomes OR maternal mental health status

Inclusion Criteria

Studies were eligible if they assessed adult women (≥18 years) with postpartum depression (PPD) or postpartum depressive symptoms. Most studies from the United States were included in this review since there is some degree of similarity in terms of how healthcare systems provide access, screening procedures, and clinical management pathways. Eligible studies were limited to epidemiological studies, studies assessing risk factors (e.g., social support and prior mental illness), studies examining screening methods of identifying individuals suffering from PPD, and studies assessing management strategies for treating affected women (e.g., psychotherapy and antidepressant use). In addition, emerging digital health interventions (e.g., telehealth or wearable technology) were also examined.

Eligible studies were limited to observational studies (cross-sectional, cohort, and case-control), as well as clinical studies and reviews relevant to the clinical care of an adult female suffering from PPD. Eligible studies must provide measurable outcomes related to the diagnosis of PPD (e.g., prevalence, level of symptom severity, examination of screening methods for PPD, or treatment decisions). In addition, studies and/or reports that included differences or limitations related to access/utilization of healthcare services for individuals of different socioeconomic statuses or racial groups will be considered in order to capture the real-world clinical and public health implications of PPD.

Study Selection Process

From all records retrieved from the database reference management software, any duplicates were removed. The titles and abstracts of all the articles identified were evaluated by two independent reviewers for eligibility, followed by a full-text evaluation of studies that potentially met eligibility criteria. In the event of any disagreements regarding decisions about eligibility, these were resolved through discussion with the other reviewer(s) or with a third reviewer. A PRISMA flow diagram was used to document the study selection process.

Quality Assessment

The methodological quality of included studies was assessed using validated tools according to study design. The Newcastle-Ottawa Scale (NOS) was applied to observational studies, including cross-sectional and cohort designs, with evaluation across three domains: selection, comparability, and outcome assessment [21]. Each study was independently assessed by two reviewers, and discrepancies were resolved through consensus. Studies were categorized as low, moderate, or high quality based on their total scores.

For randomized studies, appropriate risk-of-bias assessment tools were applied based on study design. Review articles, systematic reviews, and umbrella reviews were assessed using the Revised Assessment of Multiple Systematic Reviews (R-AMSTAR) tool, with explicit evaluation across its defined domains to ensure consistency and transparency in quality appraisal [22].

Data Extraction

A standard data extraction form was developed. Extracted variables consisted of author, year of publication, type of study, place of study in the US, number of participants, demographics, prevalence of postpartum depression (PPD), identified risk factors, identification or use of screening tools, and treatment. Other variables collected included access to healthcare, disparities in care, and use of digital health interventions. Data extraction was performed independently by two reviewers, and discrepancies were resolved through consensus. Primary studies were considered the main unit of analysis for the synthesis of epidemiological and risk factor evidence. Review articles, meta-analyses, and umbrella reviews were used to support contextual interpretation and were not treated as independent sources of primary data to avoid duplication or overrepresentation of evidence.

Results

**Figure 1:**
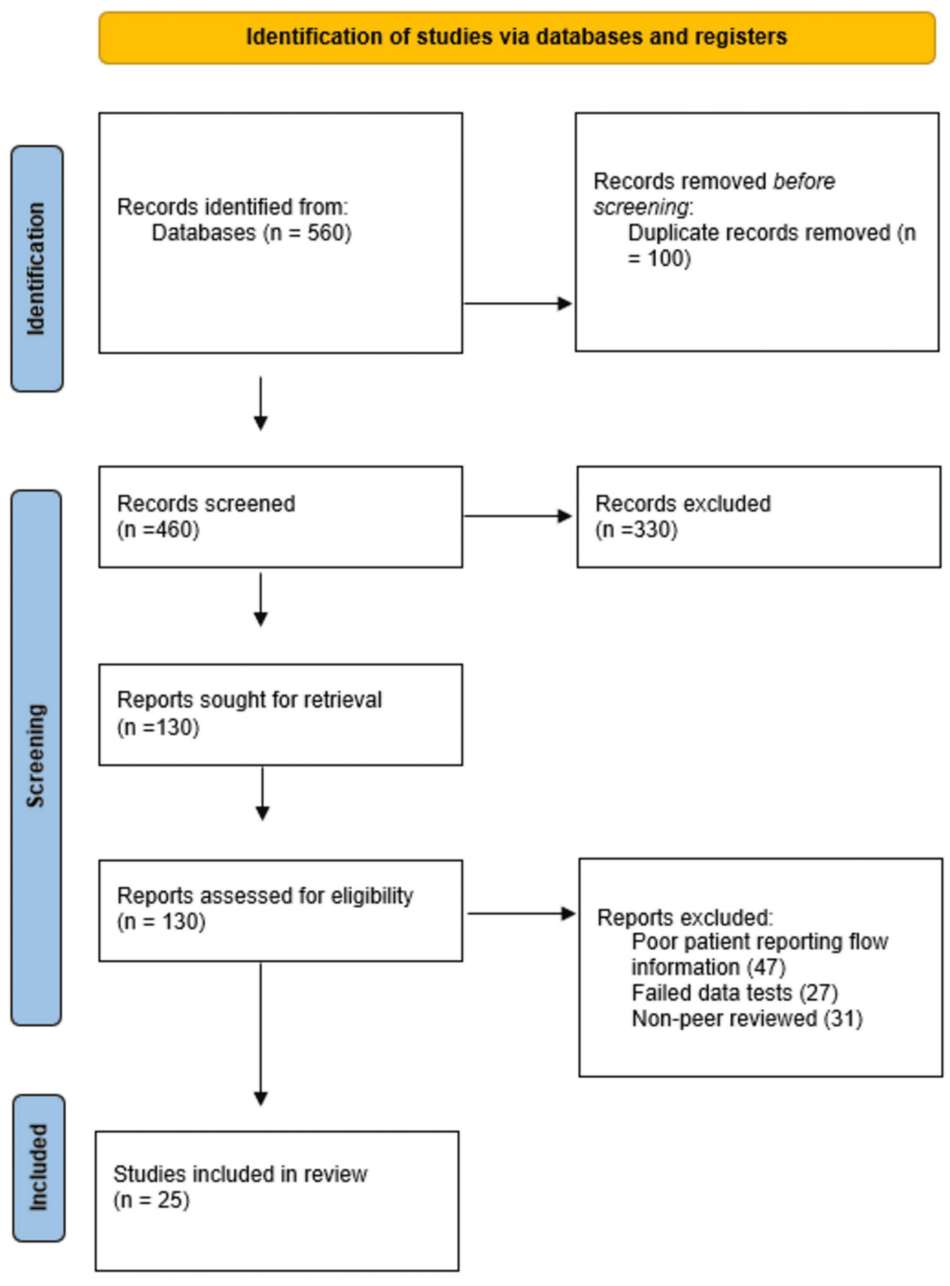
PRISMA flow diagram indicating the study selection and inclusion process This review was conducted in accordance with the PRISMA 2020 guidelines [20]. This work is licensed under CC BY 4.0. PRISMA: Preferred Reporting Items for Systematic Reviews and Meta-Analyses

The overall search across the databases yielded 560 studies. Then, 100 duplicates were removed, leaving 460 abstracts and titles to be screened. Of the studies, 330 were eliminated as they failed to meet the eligibility criteria (studies not addressing postpartum depression, population was not based in the United States, and the studies lacked relevant outcomes). Of the 130 full-text articles assessed, 105 were excluded for the following reasons: non-US population, irrelevant outcomes, not specific to postpartum depression, inappropriate study design, or insufficient data for extraction. A total of 25 studies met the inclusion criteria and were included in the final analysis (Figure 1). The 25 studies included in the study were all either observational studies or randomized trial studies conducted in the United States. They were included in the systematic review to provide a qualitative analysis.

Table 2 presents the summary characteristics of the 25 included studies.

**Table 2:** Summary of included studies NHANES: National Health and Nutrition Examination Survey, PPD: postpartum depression, BMI: body mass index, SEM: structural equation modeling, *ESR1*: estrogen receptor 1, PRAMS: Pregnancy Risk Assessment Monitoring System, RCTs: randomized controlled trials, BDNF: brain-derived neurotrophic factor, Val66Met: valine with methionine at codon 66, PTSD: post-traumatic stress disorder

Reference	Study design	Population	Outcome focus	Key findings
Gardner et al. (2025) [1]	Cross-sectional (NHANES)	US pregnant and postpartum women	Prevalence, treatment trends	Increasing trends in the diagnosis and treatment of antepartum and PPD over time.
Khadka et al. (2024) [2]	Observational study	Postpartum women by race/BMI	Disparities in PPD	Significant racial, ethnic, and BMI-related disparities in PPD prevalence.
Robbins et al. (2023) [3]	Observational study	Postpartum women	Timing of symptoms	PPD symptoms vary across postpartum periods, emphasizing the need for continuous screening.
Liu et al. (2022) [4]	Retrospective cohort	Hospital discharge data	Disparities	Socioeconomic and racial disparities are strongly associated with PPD risk.
Cho et al. (2022) [5]	Cross-sectional	Postpartum women	Social support	Strong inverse relationship between social support and PPD.
Xu et al. (2026) [6]	Umbrella review	General postpartum population	Risk factors	Identified multifactorial risks, including psychosocial, biological, and obstetric factors.
Mercier et al. (2013) [7]	Prospective cohort study	Pregnant and postpartum women	Pregnancy intention and PPD	Unintended pregnancy was associated with an increased risk of PPD symptoms.
Coburn et al. (2016) [8]	Observational longitudinal cohort study	Low-income Mexican American pregnant and postpartum women (USA; n = 269)	Postpartum depressive symptoms; psychosocial stress; role of social support	Multiple domains of stress (partner stress, family stress, daily hassles, and culture-specific stress) significantly predicted postpartum depressive symptoms.
Wan Mohamed Radzi et al. (2021) [9]	Cross-sectional (SEM)	Postpartum women	Symptom modeling	Structural relationships show that psychosocial factors influence PPD symptoms.
Gilden et al. (2020) [10]	Cohort study	Women with severe PPD/psychosis	Mother-infant bonding	Severe PPD negatively affects maternal bonding with infants.
Comasco et al. (2011) [11]	Genetic association study	Postpartum women	Postpartum depressive symptoms, BDNF polymorphism, and seasonality	The BDNF Val66Met polymorphism and season of delivery influenced postpartum depressive symptoms.
O’Connor and Su (2023) [12]	Secondary data analysis	PRAMS population	Social determinants	Social and economic determinants significantly influence PPD risk.
Ali et al. (2021) [13]	Review article	Postpartum women	Treatment (brexanolone)	Brexanolone is effective for severe PPD but requires monitoring.
Hurwitz et al. (2024) [14]	Cross-sectional	Women using wearables	Digital biomarkers	Wearables can help detect early signs of PPD.
Lewkowitz et al. (2024) [15]	Systematic review and meta-analysis	RCTs (postpartum women)	Digital interventions	Digital health interventions significantly reduce PPD symptoms.
Arias et al. (2022) [16]	Retrospective cohort	Postpartum women	Telehealth care	Telehealth improves access to postpartum care and mental health support.
Miura et al. (2023) [17]	Systematic review and meta-analysis	Postpartum women	Application-based prevention	Mobile applications show moderate effectiveness in preventing PPD.
Zhao et al. (2021) [18]	Systematic review and meta-analysis	Women with PPD	Telehealth interventions	Telehealth significantly improves depression outcomes.
Avalos et al. (2016) [19]	Cross-sectional	Pregnant and postpartum women in obstetric care	Perinatal depression screening, treatment, and outcomes	Universal screening programs improved identification, treatment uptake, and clinical outcomes for perinatal depression.
Chandra et al. (2024) [23]	Prospective study	General population	Genetic markers	Multiple genetic markers linked to increased PPD susceptibility.
Wisner et al. (2013) [24]	Observational cohort study	Postpartum women with positive depression screens	Timing of onset, self-harm thoughts, and psychiatric diagnoses	Many women with PPD reported self-harm thoughts, with varied onset timing and psychiatric diagnoses.
Nguyen et al. (2022) [25]	Pilot study	Families of postpartum women	Symptom recognition	Family members can identify early PPD symptoms.
Ayisire et al. (2022) [26]	Review article	Postpartum women	Substance use	Cannabis use may worsen or influence PPD symptoms.
Ko et al. (2012) [27]	Review article	US pregnant and non-pregnant women of reproductive age	Depression prevalence and treatment patterns	Depression was common among reproductive-age women, although many affected women did not receive treatment.
Muzik et al. (2024) [28]	Prospective study	Mothers with depression and PTSD	Parenting behaviors and maternal psychopathology	Maternal depression and PTSD negatively affected perceived and observed parenting behaviors.

Table 3 presents the methodological quality assessment of the included observational studies using the Newcastle-Ottawa Scale (NOS) [21].

**Table 3:** Quality assessment for observational studies (NOS) Quality assessment was carried out using the NOS [21]. NOS: Newcastle-Ottawa Scale

Study (first author, year)	Selection (maximum: 4)	Comparability (maximum: 2)	Outcome (maximum: 3)	Total score (maximum: 9)	Quality rating
Gardner et al. (2025) [1]	4	2	3	9	High
Khadka et al. (2024) [2]	4	2	3	9	High
Robbins et al. (2023) [3]	3	2	3	8	High
Liu et al. (2022) [4]	4	2	3	9	High
Cho et al. (2022) [5]	3	2	2	7	Moderate
Mercier et al. (2013) [7]	4	1	2	6	Moderate
Coburn et al. (2016) [8]	4	1	3	8	High
Wan Mohamed Radzi et al. (2021) [9]	3	1	2	6	Moderate
Gilden et al. (2020) [10]	3	2	3	8	High
Comasco et al. (2011) [11]	2	2	2	6	Moderate
O’Connor and Su (2023) [12]	3	2	3	8	High
Hurwitz et al. (2024) [14]	3	1	2	6	Moderate
Arias et al. (2022) [16]	4	2	3	9	High
Avalos et al. (2016) [19]	4	2	3	9	High
Chandra et al. (2024) [23]	4	2	3	9	High
Wisner et al. (2013) [24]	3	2	3	8	High
Nguyen et al. (2022) [25]	2	1	2	5	Moderate
Ayisire et al. (2022) [26]	3	1	2	6	Moderate
Ko et al. (2012) [27]	4	2	2	8	High
Muzik et al. (2024) [28]	3	2	3	8	High

Table 4 presents the methodological quality assessment of the included systematic reviews and meta-analyses using the Revised Assessment of Multiple Systematic Reviews (R-AMSTAR) tool [22].

**Table 4:** Quality assessment for review studies (R-AMSTAR evaluation) 1: a priori design, 2: duplicate study selection and data extraction, 3: comprehensive literature search, 4: publication status as an inclusion criteria, 5: list of included and excluded studies, 6: characteristics of included studies, 7: documented assessment of the scientific quality of included studies, 8: appropriate use of the scientific quality in forming conclusions, 9: appropriate use of methods to combine study findings, 10: assessment of publication bias likelihood, 11: conflict of interest documentation Quality assessment was carried out using R-AMSTAR evaluation [22]. R-AMSTAR: Revised Assessment of Multiple Systematic Reviews

Study (first author, year)	1	2	3	4	5	6	7	8	9	10	11	Score
Xu et al. (2026) [6]	1	1	1	1	1	1	1	1	1	1	1	11
Ali et al. (2021) [13]	1	0	1	1	1	1	0	1	1	1	1	9
Lewkowitz et al. (2024) [15]	1	1	1	1	1	1	1	1	1	1	1	11
Miura et al. (2023) [17]	1	1	1	1	1	1	1	1	1	1	1	11
Zhao et al. (2021) [18]	1	1	1	1	1	1	1	1	1	1	1	11

Study findings

As evidenced by this systematic review, postpartum depression (PPD) has been a major issue of public health concern in the United States since its prevalence rates have been between 10% and 20%, and have been on a rising trend in terms of diagnosis and treatment over the years [1]. The current evidence suggests that race, ethnicity, and socioeconomic status are the key determinants of the disparities in PPD, and minority and disadvantaged groups are more prone to the disease burden [2,4,12]. Also, PPD symptoms do not align at one time of the postpartum period, which demonstrates the necessity of continuous screening as opposed to a one-time evaluation [3]. PPD has a detrimental impact on the quality of life of the mother and the closeness to the infant that may disrupt the development of emotional, social, and cognitive aspects of the infant [23]. Postpartum depression and mother-to-infant bonding difficulties (MIBD) are all major problems that have been identified as highly critical to the mental health of both the mother and the child during the subsequent periods following childbirth [24]. PPD is dangerous to the life of the mother and influences the quality of childcare. Depressive symptoms should be identified early by family members [25].

There are significant clinical outcomes of PPD, such as disrupted mother-infant bonding and adverse developmental outcomes in children [10]. There are also emerging reasons according to which family members may be important in the early identification of the symptoms to enhance timely intervention [25]. Moreover, drugs, including cannabis, can influence the severity of depressive symptoms and deteriorate outcomes [26].

PPD symptoms, which are often screened using the Edinburgh Postnatal Depression Scale, comprise major to minor negative symptoms that satisfy the diagnosis criteria of depression, including poor sleep, poor concentration, irritability, mood swings, feeling of guilt, extreme sadness, indifference, anxiety, and occurrences of alterations in energy, poor appetite, and other minor symptoms of depression that are almost ignored and swept away as postpartum [26]. The results all lead to the conclusion that PPD is a multifactorial disorder that is affected by psychological, social, and biological factors. Mental illness that developed previously, unwanted pregnancy, lack of social support, and socioeconomic stressors were some of the strongest predictors of PPD [5,12,27]. Biological determinants of the condition, such as genetic susceptibility and association with certain gene markers, including *ESR1*, also support the biological causation of the condition [11,23]. In addition to this, there is solid evidence that social support is protective, which minimizes the risk and severity of postpartum depressive symptoms [5,28].

Management-wise, the conventional methods of treatment (psychotherapy and antidepressants) are still in the focus, whereas newer drugs (such as brexanolone) demonstrate efficacy in severe cases despite their practical disadvantages [13]. Notably, digital health interventions, such as telehealth, mobile applications, and wearable technologies, have demonstrated encouraging outcomes in enhancing care access, early disease detection, and symptom management [14,16,18,19]. Such strategies are especially useful when eliminating care barriers such as cost and access.

Discussion

Epidemiology and Burden of Postpartum Depression

Results of the present review demonstrate that postpartum depression (PPD) is a significant public health concern in the United States. The reported prevalence of postpartum depression varied across studies, generally ranging from 10% to 20%, depending on population characteristics, screening tools, and timing of assessment. These variations highlight differences in study design and measurement approaches across included studies [1]. These trends could indicate a better awareness and screening behaviors, but inequalities among various population groups are observed. Research has continually indicated that women with racial and ethnic minority backgrounds, and those with increased pre-pregnancy body mass index (BMI) are disproportionately impacted by PPD [2,4]. Moreover, the time of appearance of symptoms differs significantly; in some women, it appears immediately after childbirth, while in others, there is a delay in onset, thus the importance of repeated and continuous screening during the postpartum period [3]. PPD is not only a critical concern to healthcare systems and policymakers, but it also impacts the development of infants, family dynamics, and the long-term maternal mental health outcomes of the person with the condition.

Clinically, postpartum depression is best conceptualized as a multifactorial biopsychosocial condition, emerging from the interplay of biological factors (such as hormonal fluctuations and sleep disturbances), psychological susceptibilities (including previous psychiatric disorders, cognitive styles, and shifts in identity), and social determinants (such as support networks and financial stressors), in addition to obstetric factors related to pregnancy and childbirth. This multifaceted nature accounts for its diverse clinical presentations, ranging from traditional depressive symptoms to anxiety, irritability, or mixed states, and the variability in treatment outcomes. Consequently, effective management extends beyond simple symptom screening, requiring a holistic assessment that integrates psychiatric background, social circumstances, and perinatal influences to tailor comprehensive, multimodal care for each individual [5,6].

Risk Factors and Determinants of Postpartum Depression

This review has identified that PPD is a multifactorial disorder whose occurrence is caused by a combination of psychological, social, and biological factors. In line with available literature, previous mental illness has been found to be one of the most powerful predictors of PPD, along with psychosocial stressors, including financial problems, relationship issues, and unintended pregnancies [7,12]. Oftentimes, low levels of social support were found to be a significant determinant, and there was substantial evidence showing an inverse relationship between support systems and depressive symptoms [5,28]. Also, the wider social determinants, such as socioeconomic disadvantage and inaccessibility to healthcare, also play a significant role in making some populations more vulnerable [12].

Genetic and biological factors are also contributory, and research has indicated that hormonal changes after childbirth and genetic predisposition, in terms of differences in the *ESR1* gene, could be contributory factors to PPD [11,23]. Meta-analytic data also support the significance of psychosocial risk factors, especially chronic stress and the absence of emotional support, in the development of PPD [27]. These results define the necessity to be holistic in risk assessment that considers both clinical history and social context to effectively identify high-risk individuals.

Contemporary Management and Emerging Interventions

Management of postpartum depression includes a range of interventions tailored to symptom severity. Psychotherapy approaches, including cognitive behavioral therapy and interpersonal therapy, are commonly used for mild to moderate cases, while pharmacological treatment with antidepressants is considered for moderate to severe symptoms. Severe cases may require specialized interventions, including hospitalization or advanced therapies. Treatment decisions should consider breastfeeding status, comorbid conditions, and patient preferences [13]. Nevertheless, more recent pharmacological treatment options such as brexanolone have demonstrated promising efficacy in the treatment of severe cases, but they are restricted by cost and administration issues [13]. More recently, zuranolone, an oral neuroactive steroid, has been approved for the treatment of postpartum depression, offering a more accessible alternative to intravenous therapies such as brexanolone. This represents an important advancement in the pharmacological management of postpartum depression.

Notably, this review suggests a growing role of digital health interventions in care delivery; however, the evidence remains variable, and their impact on treatment outcomes may differ across settings and study designs. Telehealth has been found to promote access to postpartum care, especially to women with logistical constraints, including childcare or transportation issues [16]. Equally, mobile health applications and application-based interventions prove to be moderate in preventing and reducing PPD symptoms [17,18]. Digital screening tools and wearable technologies also provide new methods of early detection and monitoring of depressive symptoms [14,19]. While digital health interventions show promise, their implementation is associated with several challenges, including issues related to access, adherence, data privacy, and integration into clinical workflows. These tools should complement, rather than replace, standard clinical care.

Limitations

This systematic review has some limitations that are recognizable. First, the studies were a combination of observational studies, systematic reviews, and clinical trials, and this presented a heterogeneity in the study design, populations, and outcomes, and therefore could not be compared directly. Second, the majority of the evidence has been based on the US population, meaning that the results cannot be used in other foreign settings with different healthcare systems and sociocultural backgrounds. Third, the differences between the screening tools of postpartum depression and the diagnostic criteria of postpartum depression used in different research may have affected the prevalence estimates and publication of the results. In addition to this, the review may not have been complete due to the potential publication bias and non-English studies.

## Conclusions

In conclusion, this systematic review demonstrates that postpartum depression (PPD) is a significant and quite a complex social and biological problem that persists in the United States and has a complicated chain of psychological, social, and biological determinants. The findings support the necessity to detect it at the earliest possible stage, to perform frequent screening and treatment of the at-risk population. While traditional methods of treating illnesses, such as therapy and medication, are important, research has shown that technology can enhance access to care, as well as enable earlier identification of patients with depression following childbirth (PPD). Further research will need to identify ways to effectively integrate both traditional patient-centered care and innovative digital approaches to achieve positive outcomes for individuals suffering from PPD. Additionally, future studies will also need to limit variability among the available methods for accessing these useful therapies and maintain equitable access for all individuals who could benefit from this type of intervention.
